# Multimodal Phantom of Liver Tissue

**DOI:** 10.1371/journal.pone.0064180

**Published:** 2013-05-14

**Authors:** Magdalena K. Chmarra, Rune Hansen, Ronald Mårvik, Thomas Langø

**Affiliations:** 1 Department of Circulation and Medical Imaging, Faculty of Medicine, Norwegian University of Science and Technology, Trondheim, Norway; 2 Department of Medical Technology, SINTEF Technology and Society, Trondheim, Norway; 3 Department of Gastrointestinal Surgery, St. Olavs Hospital, Trondheim University Hospital, Trondheim, Norway; 4 National Center for Advanced Laparoscopic Surgery, St. Olavs Hospital, Trondheim University Hospital, Trondheim, Norway; Virginia Tech, United States of America

## Abstract

Medical imaging plays an important role in patients' care and is continuously being used in managing health and disease. To obtain the maximum benefit from this rapidly developing technology, further research is needed. Ideally, this research should be done in a patient-safe and environment-friendly manner; for example, on phantoms. The goal of this work was to develop a protocol and manufacture a multimodal liver phantom that is suitable for ultrasound, computed tomography, and magnetic resonance imaging modalities. The proposed phantom consists of three types of mimicked soft tissues: liver parenchyma, tumors, and portal veins, that are made of six ingredients: candle gel, sephadex®, agarose, glycerol, distilled water, and silicone string. The entire procedure is advantageous, since preparation of the phantom is simple, rather cost-effective, and reasonably quick – it takes around 2 days. Besides, most of the phantom's parts can be reused to manufacture a new phantom. Comparison of ultrasound images of real patient's liver and the developed phantom shows that the phantom's liver tissue and its structures are well simulated.

## Introduction

Currently, one of the fastest developing areas within medicine, both in clinical settings and in research and development, is medical imaging [1]. Medical imaging can be defined as a set of techniques that, in most of the cases, provide images of the internal parts of the body in a noninvasive manner. The term “noninvasive” means here that imaging modalities do not penetrate the skin physically. Medical imaging covers various imaging modalities, including ultrasound (US), x-ray-based methods (e.g. radiography and computed tomography (CT)), magnetic resonance (MR), nuclear medicine (e.g. positron emission tomography (PET) and single photon emission computed tomography (SPECT)), and other methods in optical imaging [1].

Medical imaging plays an important role in patients' care and is continuously being used in managing health and disease [2], [3]. For example, it is used in prevention, early detection of disease, choosing an optimal treatment, during surgical interventions, monitoring of treatment effects, etc. [3]. During surgical interventions, the imaging modality has to be readily available and preferably provide images in real-time for optimal guidance. To allow further development of image-guided therapeutic interventions and diagnostic imaging techniques and systems, phantoms that simulate human or animal tissue are needed.

Most of the commercially available phantoms are adapted for a broad market and are designed for particular applications [4]. Those phantoms are rather expensive and they are not meant to be modified or custom-fitted by the users [4]. To customize design and fabrication of the phantoms, and to overcome the above-mentioned disadvantages, various studies focus on development of techniques and ingredients to prepare tissue-mimicking materials [4]–[9].

In the literature, a multitude of techniques and tissue-mimicking materials have been proposed to prepare phantoms. The most often-used bulk matrix materials for mimicking soft tissue are based on: aqueous suspensions, agarose, gelatin, magnesium-silicate, oil gel, polyacrylamide gel, polyurethane resin, polyvinyl alcohol (PVA), polyester resin, epoxy resin, polysaccharide gels TX-150 and TX-151, polyacrylamide, and Room-Temperature-Vulcanizing (RTV) silicone [4], [5], [10]. Aqueous suspensions are the simplest tissue substitutes, in which water is used as a substitute of a tissue. Agarose- and gelatin-based tissue substitutes (also called hydrogels) are the most widely used alternatives of soft tissue that are described in the literature [4], [5]. The reasons for that are: well-characterized performance, ease of fabrication, and flexibility provided by the process that allows achieving a range of acoustic properties [4]. Reported main disadvantages of using both agarose- and gelatin-based phantoms are their lack of longevity (often limited to less than one month because of microbial invasion), and delicate structure that can easily be damaged [10]. Inclusion of biochemically toxic species prevents bacterial growth in these two tissue-mimicking materials. Oil gel-based substitutes consist of a propylene glycol, a gelatinizer, and 10 µm polymethyl methacrylate microspheres [11]. Their main advantages are: resistance to bacterial infection, and linear increase of speed of sound and attenuation with the proportion of propylene glycerol. Ethylene glycol-based oil gels, however, are not perfect substitutes of soft tissue for multimodal phantoms, because of their US characteristics; i.e. speed of sound and density are too high, and attenuation is too low. Polyurethane, polyester and epoxy resins have been reported to have good characteristics for mimicking soft tissue, including low Young's modulus, elastic recovery and immunity to bacterial infection [11]. The standardization of the polyurethane gel-based phantoms production, however, is problematic due to complex molecular design of the gels. PVA-based tissue substitutes (also referred as cryogels) have indefinite longevity, are low cost, and require a smaller amount of ingredients than the agarose- and gelatin-based tissue substitutes [12], [13]. Preparation of the PVA-based phantoms requires multiple 12-h freeze-thaw cycles and precise control of the temperature. Polysaccharide gels are used to prepare an inexpensive, conveniently moldable, and temporally stable tissue equivalent [14]. Using polysaccharide gels requires controlling gelling time by means of temperature and the ratio of the polysaccharide gel to water. Often encountered problem when making this gel mixture is incorporation of bubbles, which is a problem for US imaging. Polyacrylamide gel-based tissue substitutes are made by polymerization of the acrylamide monomer [15]. Since polyacrylamide is highly toxic, special precautions during its preparations are needed. The advantage of using RTV silicone is that the phantoms can be quickly produced [16]. Besides, RTV silicone provides a soft rubber texture similar to that of stiff tissue. The major two shortcomings of using this material are cost and hardening time.

Next to choosing bulk matrix materials, scattering particles need to be selected for optical phantoms. Often, this selection is made separately from the choice of the matrix structure. The four most common choices of scattering agents are: lipid microparticles, polymer microparticles, white metal oxide powders (including TiO_2_ and Al_2_O_3_ powders), and quartz glass microspheres. Lipid microparticles of 10 to 500 nm are biologically analogous to bilipid membranes of cells and organelles, which are believed to cause scattering in tissue. Commercially available lipid-based scatterers are milk [17], [18], fat/oil/lipid [19] and Intralipid/Nultralipid [20]–[22]. Polymer microspheres of 50 to 100 µm are produced in regular sizes, which means that repeatability and predictions of spectra are good due to well-controlled size and index of refraction [10], [23]. Wide availability of TiO_2_ powder, 20 to 70 nm, makes titanium dioxide one of the most commonly used scatterers. The key drawback of the TiO_2_ powder is that it settles when not stirred, which is a problem when fabricating aqueous suspensions. Therefore, TiO_2_ powders should be used for manufacturing gelatin- or agarose-based, RTV, and resin phantoms. The use of quartz glass microspheres (250 nm) is less established [10].

The goal of this work was to develop a protocol and manufacture a multimodal liver phantom. The main requirements for the phantom were: suitability for US, CT, and MR imaging modalities; easy production; standardized fabrication; low cost; and life-cycle environment friendliness, including re-usability of phantom's parts and materials, and avoidance of using toxic resources.

The developed protocol is a combination and modification of procedures proposed by Fredfeldt [24] and Schweiger et al. [25]. The phantom consists of three types of mimicked soft tissues: liver parenchyma, tumors, and portal veins. The main ingredient of the liver parenchyma is candle gel. To obtain homogeneous and adequate echogenicity of the parenchyma, sephadex® has been equally distributed in it. The tumors have been made of a mixture of agarose, sephadex, glycerol, and distilled water. Agarose has been chosen for its low attenuation of US beams, and to obtain a bulk-like substance. Besides, agarose is a good T2-relaxation modifier in MR imaging. Sephadex allows for obtaining homogeneous and adequate background US scattering, whereas glycerol helps to obtain adequate speed of US. Distilled water makes up the remaining volume needed for tumor tissue. Star shape cross-sectioned silicone cords have been chosen to mimic the portal veins.

## Materials and Methods

The compositions of the materials used for mimicking those soft tissues and the whole equipment needed to manufacture multimodal phantom are described below. In the section *Reagents*, essential materials used to produce the phantom are split into reagents. In *Reagent Setup*, details of composition of buffers are given. Section *Equipment* provides the reader with a description of all equipment needed. In the section *Protocol*, a detailed method to manufacture the phantom is described together with timing, critical steps, pause points, and troubleshooting.

A choice of concentrations of ingredients in both liver parenchyma and tumor tissue was obtained after a series of iterations that involved varying the concentrations of all the ingredients and assessing obtained US, CT, and MR images.

### Reagents

- Candle gel (www.panduro.com)
- Sephadex (Fine, 20–80 µm, Sephadex G2580, Sigma-Aldrich, www.sigmaaldrich.com)

‘CAUTION’ The toxicological properties of this material have not been fully investigated. May cause eye, skin, and respiratory tract irritation.

- Agarose (Type I-A, low EEO, Agarose A0169, Sigma-Aldrich, www.sigmaaldrich.com)
- Glycerol (99% GC, Sigma-Aldrich, www.sigmaaldrich.com)

‘CAUTION’ Avoid contact and inhalation: Target organ(s): Kidneys. Hygroscopic.

- Distilled water
- If required: pigments for candle gel (www.panduro.com) and/or food dyes for agarose-based mixture (http://www.wilton.com)

‘CRITICAL’ Use pigments for candle gel or food dyes for agarose-based mixture if coloration of liver parenchyma or tumors is needed. Note, adding a higher amount of pigments to candle gel will decrease the transparency of the liver parenchyma.

### Reagent Setup

#### Carpet

Cut the carpet in the form of the bottom of the phantom container.

#### Tumor tissue

The tumor tissue is made of 7.5 g of agarose, 30 ml of glycerol, 200 ml of distilled water, and 4 g of sephadex. The tumor tissue can be prepared in one week in advance and stored in the fridge.

#### Liver parenchyma

Liver parenchyma is made of 1000 g of candle gel and 4.2 g of sephadex. The parenchyma can be made months in advance and stored in congealed form at a room temperature. Before preparing a phantom, the parenchyma should be heated up (while gently stirring) until it becomes liquid. Then, it should be placed in the vacuum drying oven.

### Equipment

Note that the equipment described below is the one available in our laboratory. Nevertheless, all this equipment can be modified according to needs and preferences of the reader.

**Figure 1:**
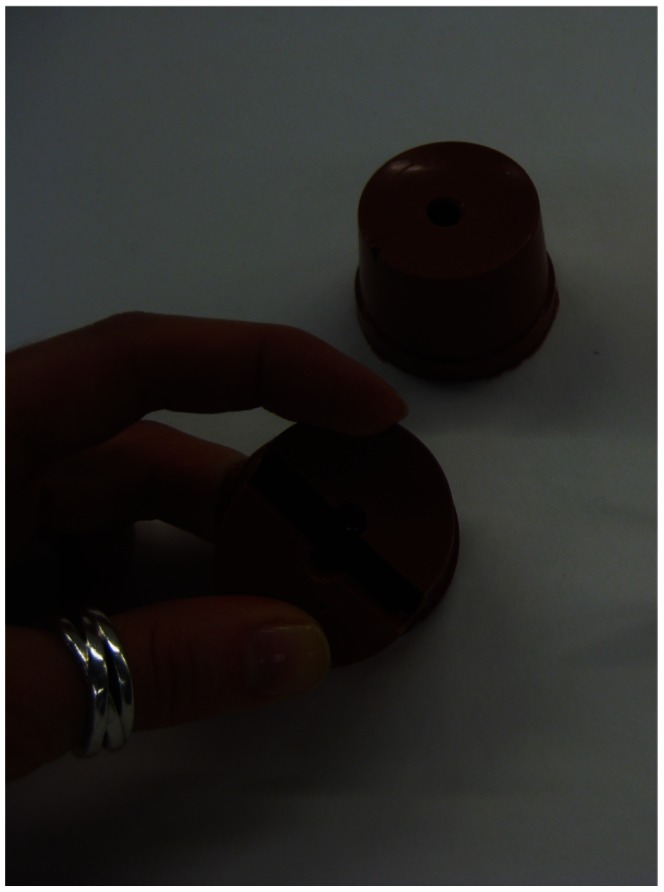
Silicone molds for manufacturing tumors.

**Figure 2:**
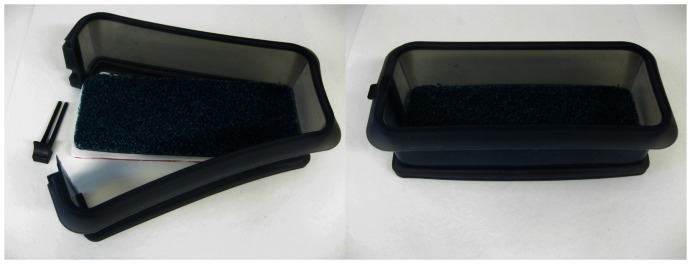
Silicone phantom container with carpet. Left: open container; Right: closed container.

- Graduated cylinder
- Erlenmeyer (conical) flask
- Beaker
- Spatulas
- Silicone molds for making tumors (tumor diameter = 10 mm, prepared at the Dept. Medical Techniques at St. Olavs Hospital, Trondheim) (Fig. 1)
- Lab scale (readability 1 mg, Sartorius, http://www.sartorius.com)
- Lab scale (readability 1 g, Sartorius, http://www.sartorius.com)
- Laboratory hot plate magnetic stirrer (MR Hei-Standard, Heidolph Instruments, http://www.heidolph-instruments.com)
- Magnetic stirrer bars
- Vacuum drying oven (B8000, Termaks, http://www.termaks.com)
- Phantom container (Lékué duo loaf spring form with removable base, duo rectangular, 24 cm, Lékué, www.lekue.es) (Fig. 2)

We recommend using a silicone mold if the phantom should easily be removed from the phantom container. Use a plastic box if the phantom should be kept in the phantom container and if it is required to, for example, apply CT or MR fiducial markers on it.

**Figure 3:**
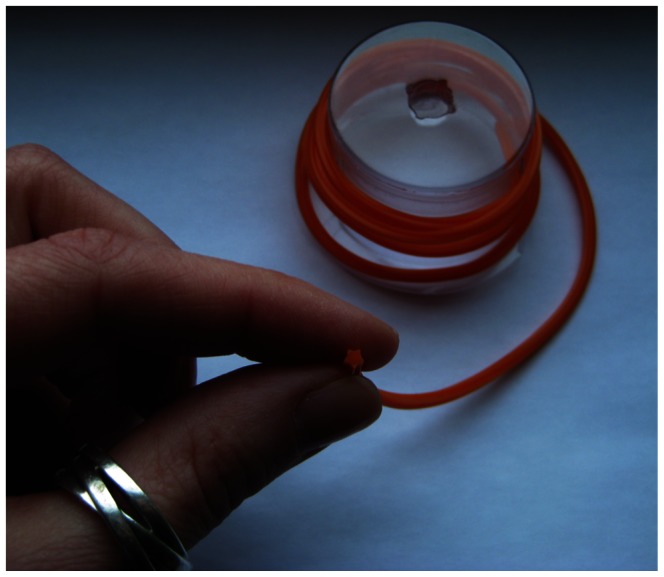
Silicone string for mimicking portal veins.

- Silicone string (internal diameter = 2 mm, outside diameter = 2.9 mm, Master Class Silicone Kitchen Twine, Kitchen Craft, www.kitchencraft.co.uk) (Fig. 3).

To mimic portal veins, we recommend using silicone string with a star-shaped cross-section.

- Carpet

We recommend using carpet to avoid specular reflections from the bottom of the phantom container and the surface on which the phantom container is placed.

## Procedure

**Preparation of the Phantom Container**

**TIMING∼1 day.**

1. Place the carpet in the phantom container (Fig. 2).
2. Pour hot water onto the carpet (just to cover the whole carpet), cover the phantom container, and leave it for approximately 24 hours.

‘CRITICAL STEP’ Air bubbles left in the carpet cause reflections in the ultrasound images. They also cause development of air bubbles in the phantom's parenchyma. Remove all air bubbles from the wet carpet by gently wiping carpet's surface with fingers.

**Preparation of the Tumor Tissue**

**TIMING∼2 h.**

1. Place agarose, distilled water and glycerol in a conical flask together with a stirring magnetic bar.
2. Place the conical flask on the hot plate magnetic stirrer and heat it up under magnetic steering at the maximum speed of 250 rotations/minute.

‘CRITICAL STEP’ Ensure that stirring the mixture does not promote development of air bubbles.

1. Boil the agarose mixture for around 2 minutes.
2. Add sephadex (while stirring).

‘CRITICAL STEP’ Distribute sephadex in a small quantity of the agarose mixture first. Once the sephadex is equally distributed, pour the mixture to the rest of the agarose mixture.

‘CRITICAL STEP’ Ensure that stirring the mixture does not promote development of air bubbles. The stirring speed, however, should be sufficiently high to ensure that high-density sephadex does not settle.

1. If required: add pigments while gently stirring the mixture.
2. Cool the agarose mixture to around 40°C while continuously stirring.
3. Pour the agarose mixture into the tumor molds.
4. Place the tumor molds in the fridge for at least 0.5 hour.

‘PAUSE POINT’ Can be left up to one week in the fridge.

1. Remove the agarose-based tumors from the tumor molds.

‘CRITICAL STEP’ It is recommended not to keep the agarose-based tumors for longer than one hour in the room temperature.

‘TROUBLESHOOTING’ Troubleshooting advice can be found in Table 1.

**Table 1:** Troubleshooting.

Step	Problem	Possible reason	Solution
11	The shape of tumor does not resemble mold's cavity	Too thick agarose-based mixture	Repeat the preparation of the tumor tissue. In step 8, pour the mixture into the molds when it has a higher temperature than 40°C
20	Big air bubbles	Air bubbles left in the carpet	Use spatula to gently move the air bubbles towards phantom container's walls
		Air bubbles introduced during preparing candle gel mixture	Use spatula to gently move the air bubbles towards phantom container's walls. Place remaining candle gel mix in the vacuum drying oven for 0,5 h
		Air bubbles introduced during pouring candle gel mixture	Use spatula to gently move the air bubbles towards phantom container's walls. Reduce the speed of pouring the gel in the phantom container
24	Big air bubbles	Air bubbles introduced during preparing candle gel mixture	Use spatula to gently move the air bubbles towards phantom container's walls. Place remaining candle gel mix in the vacuum drying oven for 0,5 h
		Air bubbles introduced during pouring candle gel mixture	Use spatula to gently move the air bubbles towards phantom container's walls. Pour successive candle gel layers via the surface of the phantom container's wall. Reduce the speed of pouring the gel in the phantom container
		Air bubbles from the surface of the tumors	Use spatula to gently move the air bubbles towards phantom container's walls. Consider making new tumors
	Small air bubbles	Air bubbles introduced during pouring candle gel mixture	Use spatula to gently move the air bubbles towards phantom container's walls. Pour successive candle gel layers via the surface of the phantom container's wall
		Air bubbles from the surface of the tumors	Use spatula to gently move the air bubbles towards phantom container's walls. Consider making new tumors

**Preparation of the Liver Parenchyma**

**TIMING∼3.5 h.**

1. Place candle gel together with a magnetic stirring bar in a beaker.
2. Place the beaker on the hot plate magnetic stirrer and heat it up until candle gel becomes liquid (around 80–90°C).
3. Distribute sephadex in the liquid candle gel.

‘CRITICAL STEP’ Distribute sephadex in a small quantity of the candle gel first. Once the sephadex is equally distributed, pour the mixture to the rest of the candle gel. Use magnetic stirring to equally distribute the mixture in the candle gel.

1. Place the candle gel mixture in the vacuum drying oven (at around 80–90°C) for at least 2 hours.

‘CRITICAL STEP’ This step is recommended in order to remove air bubbles from the candle gel mixture.

‘PAUSE POINT’ Candle gel mixture can be left overnight in the vacuum drying oven (at around 80–90°C).

1. Place the candle gel mixture on the hot plate magnetic stirrer and gently stir it (at maximum speed of 250 rotations/minute) for about 1 minute.

‘CRITICAL STEP’ High-density sephadex settles down when the candle gel mixture is kept in the vacuum drying oven. Stir gently the mixture to equally distribute sephadex in it.

1. If required: add pigments while gently stirring the mixture.

**Preparation of the Phantom**

**TIMING∼12 h.**

1. Remove the excessive amount of water from the phantom container.
2. Pour a thin layer of the candle gel mixture into the phantom container.

**Figure 4:**
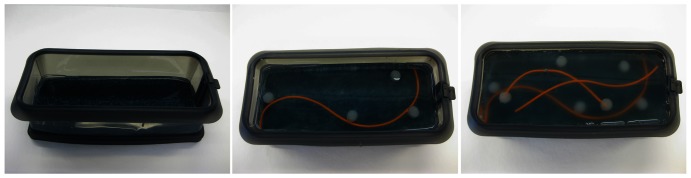
Manufacturing multimodal phantom. Left: step 19 of the protocol; Middle: step 22 of the protocol; Right: the end result of the protocol – multimodal phantom mimicking liver tissue.

‘CRITICAL STEP’ Cover the whole carpet with the candle gel mixture (Fig. 4). This will lock the remaining air bubbles in the carpet and will avoid their distribution into the next layers of the candle gel mixture.

‘CRITICAL STEP’ Gently pour the candle gel mixture into the phantom container from one corner. The candle gel mixture will spread itself on the surface without introducing additional air bubbles.

1. Use a spatula to remove any air bubbles that have been introduced during step 19.

‘CRITICAL STEP’ Remove the air bubbles when the candle gel mixture (liver parenchyma) is still liquid. It is not recommended to remove air bubbles when the candle gel mixture is half-congealed, as in most of the cases it will result in expanding the air bubbles.

‘CRITICAL STEP’ Remove the air bubbles by gently moving them towards phantom container's walls.

‘TROUBLESHOOTING’ Troubleshooting advice can be found in Table 1.

1. Place the phantom in the fridge for around 15 minutes.
2. Remove the phantom from the fridge and place agarose-based tumors and/or silicone blood vessels on the liver parenchyma (Fig. 4).
3. Pour a layer of the liver parenchyma carefully, trying not to introduce air bubbles.

‘CRITICAL STEP’ Pour the candle gel mixture into the phantom container from one corner. The candle gel mixture will spread itself on the surface without introducing additional air bubbles.

‘CRITICAL STEP’ Do not make too thick layers, as they will cause longer congelation time and will keep higher temperature of the phantom for a longer time. This might cause changes in forms of the agarose-based tumors. The best results are obtained with the layers' thickness of 1–2 cm.

1. Use a spatula to remove the air bubbles that have been introduced during step 22.

‘CRITICAL STEP’ Remove the air bubbles when the candle gel mixture (liver parenchyma) is still liquid. It is not recommended to remove air bubbles when the candle gel mixture is half-congealed, as in most of the cases it will result in expanding the air bubbles.

‘CRITICAL STEP’ Remove the air bubbles by gently moving them towards phantom container's walls.

‘TROUBLESHOOTING’ Troubleshooting advice can be found in Table 1.

1. Place the phantom in the fridge for about 15–30 minutes.
2. Repeat steps 22 to 25 as long as needed, depending on the design of the phantom, e.g. the position (height) of the tumors and blood vessels in the parenchyma.
3. Place the phantom in the fridge for at least 6 hours.

## Results and Discussion

**Figure 5:**
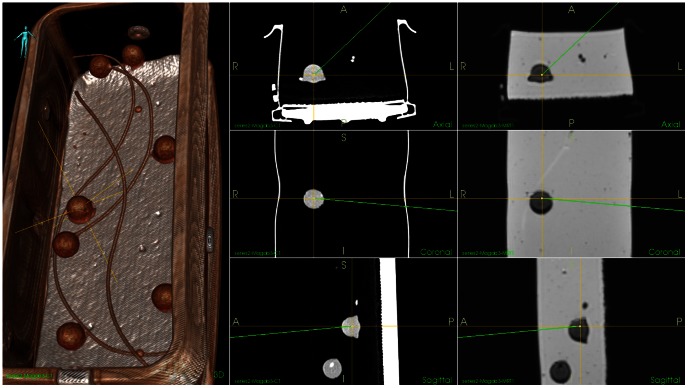
Visualization of CT and MR images of the phantom. Left: 3D volume rendering of the CT data with low-level threshold to remove the “parenchyma” (candle gel component). Middle column: Orthogonal slices through the CT volume at the position indicated by the yellow cross in the 3D rendering, axial, coronal and sagittal slices (top to bottom). Right column: Corresponding MR slices from the MR volume data at the same position in the phantom. No thresholding has been applied to the MR data.

**Figure 6:**
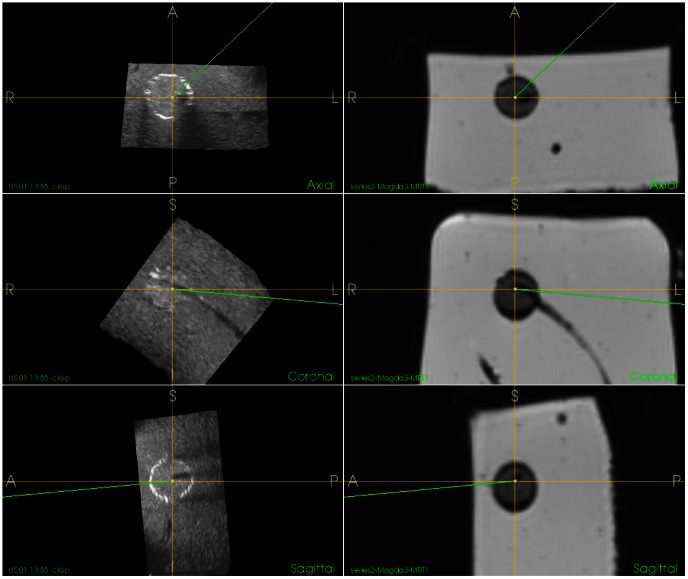
Orthogonal slicing through a 3D ultrasound volume and the MR volume data. From top to bottom: axial, coronal and sagittal slices. The tumor model has a silicone string going into it, representing a portal vein, as can be seen in the coronal slice in both ultrasound and MR.

Figure 4 presents a phantom developed using above described procedure. The US, CT and MR images of the phantom are shown in Fig. 5 and Fig. 6. The following protocols were used to scan the phantom:

- CT: liver, slice thickness 1 mm, distance between slices 0.5 mm
- MR: T1, slice thickness 1 mm, distance between slices 1 mm.

Ultrasound images were obtained using a laparoscopic ultrasound probe (OL531, Hitachi, Japan). As seen in the figure, sephadex is equally distributed in the candle gel. Besides, there are no visible borders between the layers of the liver parenchyma.

**Figure 7:**
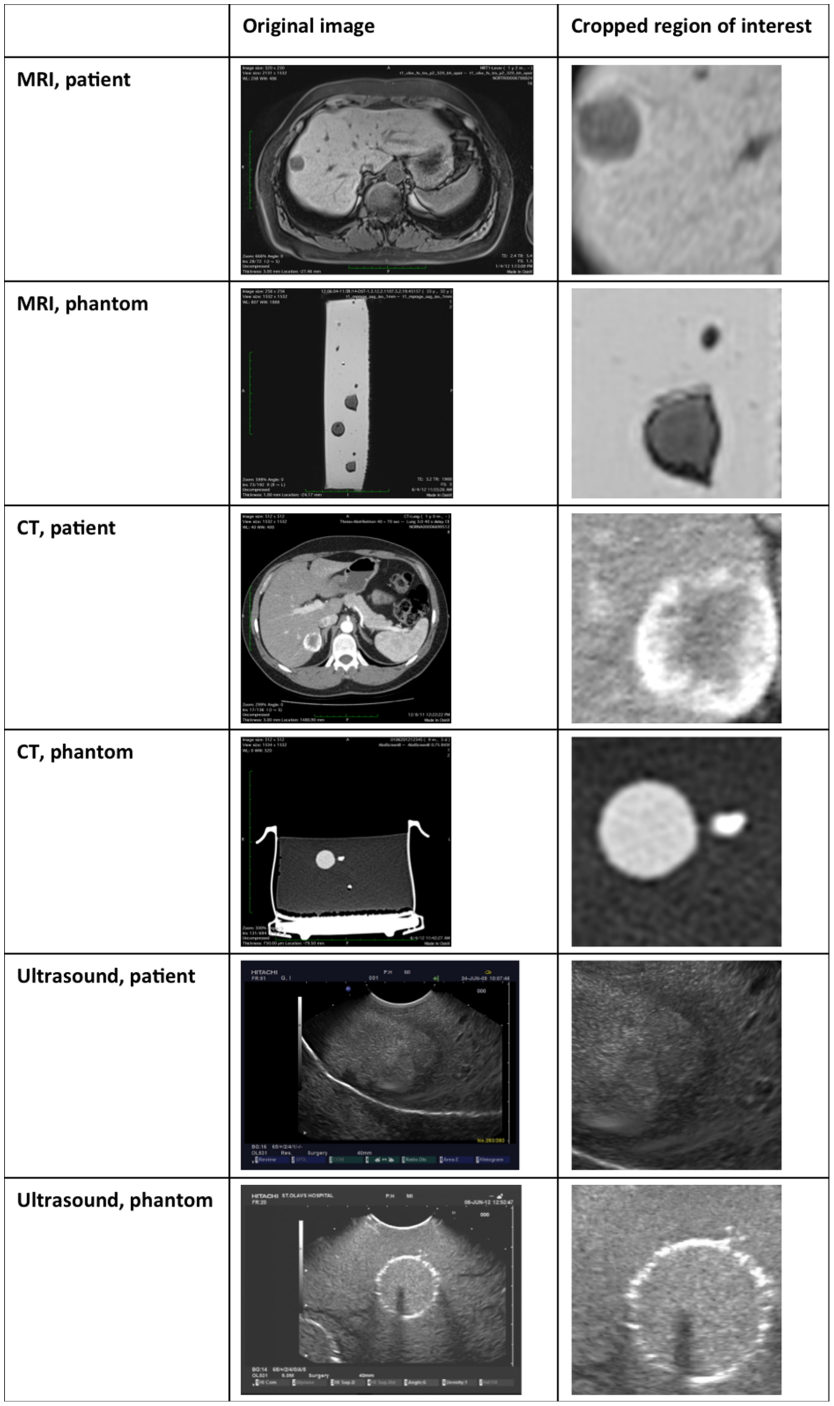
The original MR, CT, and US images from the phantom and patients along with the cropped regions.

The liver model has been positively evaluated by four expert surgeons who have done a high number of liver resection, both open and laparoscopically, radiologists, and engineers who work on medical imaging. Comparison of ultrasound images of real patient's liver [26] and the developed phantom shows that the phantom's liver tissue and its structures are well simulated. Also CT and MR images show that simulated tissues are similar to those of a real patient [26]. The original images from the phantom and patient data along with cropped regions are shown in Fig. 7. The patient sample images (MR, CT, and US) are from a patient with a metastasis in the liver. The MR imaging protocol used was a T1 weighted liver protocol.

The proposed protocol allows manufacturing multimodal phantoms that have reusable parts. Apart from tumors, both liver parenchyma and portal veins can be reused to fabricate a new phantom. For that, the “old” phantom needs to be disassembled. Disassembling of the phantom is rather easy, since removing of the agarose-based tumors and silicone portal veins is an effortless process.

Removed silicone portal veins should be cleaned before using them in a new phantom. For that, hot water can be used. The candle gel based liver parenchyma can be stored in the congealed form at a room temperature. To reuse the liver parenchyma, it is necessary to heat it up until it is liquefied.

The protocol has been developed such that it permits modifications of the phantom's content. For example, manufacturing phantoms for developing methods to identify micro-calcifications in healthy tissues requires replacement of the agarose-based tumors and silicone portal veins by calcium particles of 40–190 µm. This is useful for research on ultrasound-based diagnostics in breast.

The limitations of our protocol are related to the challenges of incorporating exact representations of vessels as different types, e.g. portal and hepatic veins, are portrayed slightly different in US images. Further research is required to refine and enable the representation of various lesions in organs in our phantom/protocol. Our protocol can be used to represent some typical lesions, but not all of them.

The developed protocol was meant for manufacturing phantoms for research purposes that include work on early diagnoses of diseases (including new methods for identifying micro-calcifications in healthy tissue [27]), evaluation of and work on navigation system (CustusX [28]), development of training setups for acquiring US skills and training and validation of navigated ultrasound in laparoscopic surgery. The advantage of using phantoms is that they are designed to mimic tissue characteristics that include acoustic properties, dimensions, and internal features. As a consequence, the phantoms can provide users with a simplified and standardized environment. There are, therefore, various purposes for which multimodal phantoms can be used. Those include:

- Initial tests of designed imaging systems
- Characterization and optimization of existing imaging systems
- Calibration and routine quality control of imaging systems
- Comparison of performance between imaging systems
- Establishment of appropriate training for translation of new imaging technologies in the clinical practice
- Training means for surgeons before performing live surgery such as US-guided puncture of lesions (e.g. biopsy or placement of radio frequency needle)
- Training means for surgeons to learn to navigate in 3D volumes
- Training means for novices to learn US tissue characterization and US anatomy
- Optimization of existing imaging technologies for clinical practice
- Testing in development of navigation systems targeted for various clinical applications, where the phantom can be tailored to fit the target organ(s)
- Assessment of 3D US acquisition and reconstruction based on knowledge about sizes and shapes of the phantom constituents both from the production and from CT and/or MR images.
