# Correction: Multimodal Phantom of Liver Tissue

**DOI:** 10.1371/annotation/b148461a-eb97-43c9-a44c-f97e7eab1711

**Published:** 2014-01-06

**Authors:** Magdalena K. Chmarra, Rune Hansen, Ronald Mårvik, Thomas Langø

The procedure in the Materials and Methods was misnumbered during the production process. The correctly numbered procedure can be viewed here: http://www.plosone.org/corrections/pone.0064180.001.cn.docx 

